# Genetic incidental findings: autonomy regained?

**DOI:** 10.1038/gim.2013.104

**Published:** 2013-08-01

**Authors:** Effy Vayena, John Tasioulas

**Affiliations:** 1 Institute of Biomedical Ethics, University of Zurich, Zurich, Switzerland; 2 Faculty of Laws, University College London, London, UK

The intense controversy sparked by the recent American College of Medical Genetics and Genomics (ACMG) recommendations^1^ on genetic incidental findings is hardly surprising or unwarranted. The recommendations offer concrete proposals on a topic that has been debated in the context of research^2,3^ but that has received inadequate attention in clinical medicine. They set out a bold, new vision of how to handle genetic information. However, they have attracted a critical backlash, largely because they deny patients undergoing sequencing a choice as to whether or not to receive a minimum list of incidental findings. Moreover, the ACMG recommendations were presented, and have been widely perceived, as generating a conflict between the values of patient autonomy and beneficence. Naturally, any proposed sacrifice of patient autonomy merits serious debate.

The major criticism brought against the recommendations is that they unacceptably override patients' consent, and hence their autonomy, which is the chief value protected by informed consent procedures. On this view, patients are wrongly denied the choice of an analysis confined to the “target” genes, and their “right not to know” is violated when they are informed by the clinician of any incidental findings. This departure from the established informed consent regime has been condemned as a disconcerting throwback to the era of medical paternalism.^4^

The ACMG working group sought to preempt these autonomy-based concerns by means of two arguments. First, that respecting patient preferences in the same manner as in targeted testing is unduly burdensome in terms of the costs of genetic counseling and the need for laboratories to mask the informatics analysis of specific genes or to ignore findings of potential medical significance (p. 568). The second argument appeals to a fiduciary duty to benefit patients by providing them with medically actionable data of the kind contained in the minimum list. According to the ACMG report, the duty to prevent harm “supersedes concerns about autonomy” (p. 568).^1^

Setting aside the question of whether the ACMG recommendations conform to established medical practice, we focus instead on the more fundamental ethical question of whether they are ultimately justifiable. In addressing this question, we wish to challenge an assumption that is apparently shared by both the ACMG working group and its critics, i.e., that the recommendations entail a conflict between a duty of beneficence toward patients, on the one hand, and respect for their autonomy, on the other. Challenging the assumption of a value conflict involves appreciating how the value of autonomy is shaped and constrained by considerations of psychological and institutional realism and also by evaluative concerns.

To begin with the evaluative constraints: the value of autonomy concerns the ability to shape the contours of one's life by making a choice from a menu of worthwhile options without undue interference from others.^5^ One does not enhance a person's autonomy by providing him or her with additional morally bad or worthless options. Of course, autonomy demands that whether or not the person takes up worthwhile options is a matter of their own free choice, but we can only speak of autonomy when such options are present. In this view, autonomy is not simply a matter of acting on one's preferences, even one's informed preferences. Therefore, proper regard for patients' autonomy cannot be a straightforward matter of enabling them to fulfill their preferences or their subjective “values.”^6^ For example, there is no sound autonomy-based argument requiring people to receive medical treatment tailored to preferences that reflect immoral (e.g., racist) or unreasonable (e.g., astrological) beliefs. This is because fashioning health-care options in this way would not contribute to their value.

If, however, autonomy involves choice from a range of worthwhile options, we must ask what value there is in patients being able to choose not to have incidental findings investigated and disclosed when undergoing sequencing. The mere existence of a preference to this effect is insufficient to generate an autonomy-based argument to provide such an option. In our view, critics of the proposals have not done nearly enough to show why such an option is valuable, let alone that its value to patients generates a right to that option. Indeed, it is arguable that the proposed ACMG regime for incidental findings actually enhances patient autonomy. It does this by generating a fuller menu of worthwhile options from which patients can make life-shaping (including life-saving) choices. The short list of conditions that must be investigated under the recommendations has been drawn up according to criteria—such as disease seriousness, high probability of onset, and medical actionability—that will reliably generate valuable options. These options are principally valuable in enabling the pursuit of improved health outcomes for the patients themselves. But they are also valuable insofar as they benefit the patient's relatives and serve the common good of promoting a healthy society.

Does this defense of the recommendations extend to the case of children? Is the child's future autonomy sacrificed by the unavailability to them or their parents of an option to undergo purely targeted testing? The onset of the overwhelming majority of conditions on the ACMG list can occur in childhood. Therefore, findings regarding these conditions (e.g., mutation at the Von Hippel–Lindau locus associated with Von Hippel–Lindau syndrome) are potentially crucial in enhancing medical options during childhood and adolescence.^7^ Decision making about minors' medical care is strongly influenced by the beliefs, values, and abilities of their parents, their clinicians, and gradually of minors themselves as their decision-making capacities evolve. Acting in a child's best interest, which centrally includes enabling them to mature into an autonomous agent, requires the availability of worthwhile options. Creating such options is hardly an affront to their future autonomy.

What about the remaining conditions on the ACMG list whose onset occurs in adulthood? Although this is a complex issue that we do not purport to resolve here, we nonetheless believe that again no violation of the child's autonomy need occur. Some of the most valuable options in people's lives involve deep personal relationships with others, especially family members. Therefore, the autonomy of the child can be served precisely through serving the well-being and autonomy of these other people. Hence, if genetic incidental findings relating to adult-onset conditions can have a bearing on the health of parents and siblings of children, their disclosure may be plausibly viewed as enhancing the autonomy of the child. Of course, in a fuller treatment, this line of argument would need to be nuanced in various ways. But consider the incongruity of telling a bereaved child that information that could have saved his or her mother's life was not sought out of respect for the child's own autonomy. With adequate counseling and other safeguards in place, it is far from obvious that a due respect for autonomy requires us to countenance such a tragic outcome.

We turn now to the need for psychological and institutional realism in understanding autonomy. The ACMG recommendations have attracted severe criticism for noncompliance with standard informed consent procedures. However, imposing such procedures in this context arguably involves unrealistic assumptions about both the capacity of patients to grasp such information and their ability to use it in arriving at reasoned decisions.^8^ It also makes implausible assumptions about the capacities of institutions to provide comprehensive and accurate information, especially given the rapid pace of developments in genetics (a consideration registered in the ACMG working group's first argument mentioned above). It is important to note that calls to adapt our demands on informed consent to a realistic assessment of the capabilities of individual agents and institutions have already emerged in the related context of genetic research.^9,10^ The question of whether standard informed consent procedures are effective in serving the value of autonomy is, however, more generally pertinent in medical genetics practice. Some of the proposals generated in relation to the research context offer alternative paradigms for handling genetic data, such as the stewardship model and the creation of trustworthy institutions. In none of these proposals is the value of consent denied, but it is not made to operate as autonomy's sole line of defense.

The ACMG recommendations are in tune with these broader developments reflecting the limitations of informed consent regimens. Under the recommendations, patients' consent figures at two crucial points: they have a choice of whether to undergo clinical sequencing at all, and they have a choice of whether to follow up on any incidental findings by undergoing medical treatment. However, as a matter of the practical workability of the proposals, we believe that it may also be advisable for patient choice to be introduced at an intermediate stage. The proposals contemplate that the clinician “contextualize any incidental findings for the patient in light of personal and family history, physical examination, and other relevant findings”^1^ (p. 567) in the course of a clinician–patient interaction that involves dialogue about the best way forward for the patient. Given the widespread sensitivity in society about genetic data, one manifested not only in lay people's attitudes but also in laws and other official pronouncements, the effectiveness of the scheme might be enhanced by an amendment. Clinicians, in the process of shared decision making, should also standardly offer patients the opportunity to opt out of receiving the details of the incidental findings generated in their case, as opposed to being informed of the general fact that such findings exist. It is worth noting, this opt-out stems not from the patient's supposed “right not to know” but from a pragmatic acknowledgment of the fact that the effective operation of the scheme may depend on making this concession to patient choice.

As genetics increasingly comes to pervade medicine, it is important to foster the trustworthiness of the mechanisms through which it finds clinical application. It is an illusion to suppose that such trust can exclusively be achieved through discrete acts of informed consent by patients, a sort of supermarket model according to which I have consented to pay for each item in my basket by putting it there. For trust to be well-placed about the items on offer, they must be of sufficient quality to be on the market, and an accountable regulatory system is necessary to ensure that this is the case.

We have argued against presenting the ACMG's recommendations as sacrificing patient autonomy to beneficence, even if this sacrifice is treated as ultimately justified. This is not only questionable as a matter of moral reasoning, but it also needlessly fuels anxieties about autonomy and trust, as the predictable critical response to the recommendations has vividly demonstrated. On the reframing of the debate advanced here, the recommendations should be defended as measures that enhance both autonomy and trust in the context of genetic medicine.

## Disclosure

The authors declare no conflict of interest.
